# Piezoresistive Tactile Sensor Discriminating Multidirectional Forces

**DOI:** 10.3390/s151025463

**Published:** 2015-10-02

**Authors:** Youngdo Jung, Duck-Gyu Lee, Jonghwa Park, Hyunhyub Ko, Hyuneui Lim

**Affiliations:** 1 Department of Nature-Inspired Nanoconvergence System, Korea Institute of Machinery and Materials, Daejeon 34103, Korea; E-Mails: yjung@kimm.re.kr (Y.J.); educk9@kimm.re.kr (D.-G.L.); 2 School of Energy and Chemical Engineering, Ulsan National Institute of Science and Technology (UNIST), Ulsan 44919, Korea; E-Mails: myidx1212@unist.ac.kr (J.P.); hyunhko@unist.ac.kr (H.K.)

**Keywords:** tactile sensor, piezoresistive, shear force, carbon nanotube, interlocking microdome, multidirectional detection

## Abstract

Flexible tactile sensors capable of detecting the magnitude and direction of the applied force together are of great interest for application in human-interactive robots, prosthetics, and bionic arms/feet. Human skin contains excellent tactile sensing elements, mechanoreceptors, which detect their assigned tactile stimuli and transduce them into electrical signals. The transduced signals are transmitted through separated nerve fibers to the central nerve system without complicated signal processing. Inspired by the function and organization of human skin, we present a piezoresistive type tactile sensor capable of discriminating the direction and magnitude of stimulations without further signal processing. Our tactile sensor is based on a flexible core and four sidewall structures of elastomer, where highly sensitive interlocking piezoresistive type sensing elements are embedded. We demonstrate the discriminating normal pressure and shear force simultaneously without interference between the applied forces. The developed sensor can detect down to 128 Pa in normal pressure and 0.08 N in shear force, respectively. The developed sensor can be applied in the prosthetic arms requiring the restoration of tactile sensation to discriminate the feeling of normal and shear force like human skin.

## 1. Introduction

The skin is the largest sensing organ that humans have and versatile in detecting tactile stimulations. While typical touch sensors installed on the screen panels of mobile devices are only dense arrays of sensitive on-off switches, human skin consists of mechanoreceptors that can discriminate the magnitude and type of external touch inputs as well as provide critical information in recognizing contacted objects and manipulating hands, feet, and other parts of human body safely.

As the demands for robots working in human-interactive way and the prosthetic arms or legs for patients have increased, there have been great interests in the development of tactile sensors capable of measuring external stimulations as human skin does. Various types of tactile sensors have been attempted based on the measurement of changes in capacitance [1,2,3,4,5,6], resistance [7,8,9,10], piezoelectric/triboelectric output [11,12,13,14], and optical signal [15,16,17]. Enhancing sensitivity and increasing flexibility of tactile sensors have become major research points in improving the performance of tactile sensors. Recently, enhanced sensitivities have been demonstrated in a capacitive tactile sensor with the microstructured rubber dielectric layers [6], resistive pressure sensors based on elastic hollow-sphere microstructured conducting polymer [18] or interlocking microstructures [19,20], and a piezoelectric pressure sensor with microstructured elastomer layer [11]. New fabrication methods such as contact printing or transfer printing enabled the development of ultra-thin and ultra–lightweight flexible tactile sensors [21,22,23,24,25]. Another research approach in improving tactile sensors has been to develop tactile sensors with multi-axis sensing capability. Various types of multi-axis sensing tactile sensors have been developed with multiple strain gauge embedded in elastomer [26,27], layered microfluidic channels [28,29], impedance-sensing electrodes with pressure transducer [30], and multiple capacitors [1,5]. However, unlike human skin equipped with distinct types of very sensitive mechanoreceptors responsible for their assigned tactile stimulations and the transduced tactile information conveyed along to central nerve system [31], most multi-axis tactile sensors were based on mapping or complicated post signal processing of sensing data from multiple sensing elements in discriminating the force direction and magnitude.

Previously, we reported on the direction sensitive and stretchable tactile sensor which has shown great sensitivities to external tactile input with the microdome structured nanocomposite piezoresistive sensing elements [32]. For the multidirectional detection, output signals from one sensing element need to be compared and analyzed with those of neighboring sensing elements to differentiate them as separated directional components. In this paper, we present a piezoresistive type tactile sensor capable of discriminating the direction and magnitude of tactile stimulations without further signal processing. With a flexible core and four sidewall structures of elastomer having embedded highly sensitive interlocking piezoresistive type sensing elements, the newly designed piezoresistive tactile sensors discriminated the magnitude and direction of the applied force successfully without complex post signal processing even under gentle touch.

## 2. Working Principle

The proposed sensor was designed to measure both magnitude and direction of the applied force without complicated post signal processing. Inspired by the organization and function of mechanoreceptors in human skin, the sensor consists of inhomogeneous structural components and multiple sensing elements.

**Figure 1:**
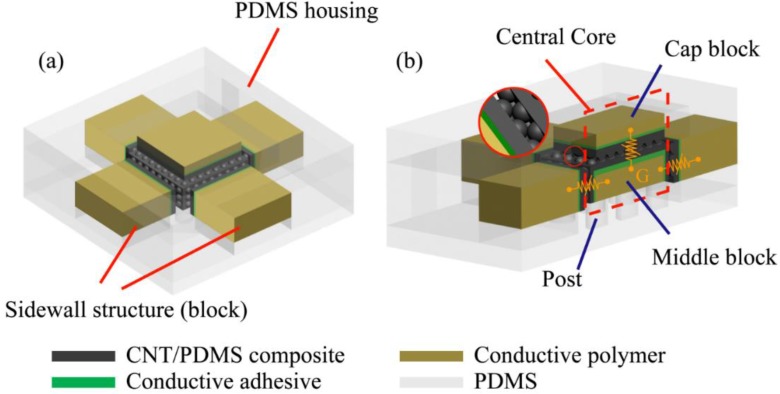
3D Schematics of the proposed sensor: (**a**) five sensing elements, one for normal force and four for shear force, are embedded to measure the magnitude and direction of applied forces. The central core is surrounded by four sidewall structures; (**b**) external force changes the contact area between two CNT/PDMS composite films. The CNT/PDMS composite films are physically and electrically connected with conductive polymer by conductive adhesive. Conductive polymer blocks act as electrodes. “G” means that the middle block acts as a common electrical reference.

Figure 1 shows the 3D schematic and working principle of the proposed sensor design. The proposed sensor consists of five basic sensing elements, and each sensing element is made of two flexible composite films interlocking each other. The composite films have microdome structures on the facing surfaces to enhance the sensitivity of each sensing element. The composite film is made of carbon nanotube (CNT) mixed polydimethylsiloxane (PDMS) and the resistance of interlocking composite films changes drastically as the contact area between two composite films varies under external forces applied perpendicular to the surface. By monitoring the resistance changes of five sensing elements, the direction and magnitude of the applied force on the sensor can be measured.

The working part of the proposed sensor can be divided into a central core and four sidewall structures. The central core is composed of stacked double blocks on a bottom housing. The bottom housing is made of PDMS and consists of micrometer scale flexible vertical posts to enable much larger movement of the whole central core in X- and Y-direction compared to Z-direction upon external shear force on X-Y plane. A middle block is made of conductive polymer and in the shape of rectangular cuboid. The middle block conductive polymer functions as a reference electrode for all sensing elements. Compared to the vertical post in the bottom block, the middle block was designed to be much stiffer, thus its deformation is much smaller than the overall movement of the central core upon a shear force. Thin CNT/PDMS composite films having microdomes are attached on the five faces of the cuboid except the bottom face which contacts the flexible posts in the bottom housing. A cap block is also made of conductive polymer. While the middle block was designed to have enough surface area on the four sidewalls to attach sensing elements measuring shear forces, the cap block was designed to measure only normal force, thus its overall thickness is much less than the middle block. A thin CNT/PDMS composite film is attached on the bottom face of the cap block such that it constitutes a normal force sensing element along with the thin film on the top face of the middle block.

Four sidewall structures are designed in the cuboid shape. They surround the central core and each sidewall structure is positioned to be parallel to the sidewalls of the middle block in the central core. Each sidewall structure is made of conductive polymer having an attached thin CNT/PDMS composite film. As similar to that of normal force sensing element, a pair of thin films, one on the sidewall structure and the other on the middle block, constitutes a shear force sensing element. Total four shear force sensing elements are designed.

The central core and four sidewall structures are assembled together in PDMS housing. The PDMS housing provides protection of sensing elements while ensuring the flexibility of the sensor.

## 3. Fabrication

**Figure 2:**
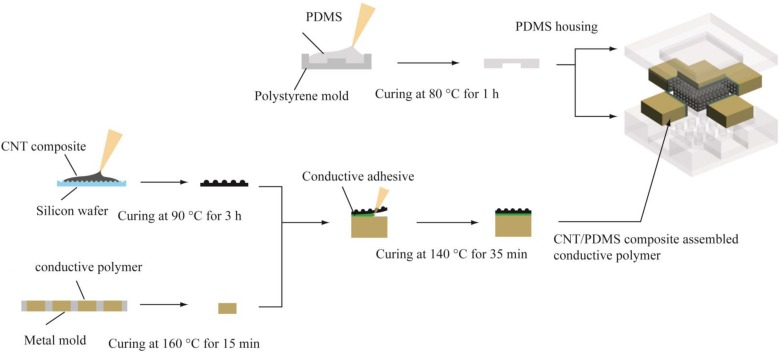
Fabrication process flow of the proposed sensor: CNT/PDMS composite film is fabricated on micromachined silicon wafer to have microdome structures and attached on a conductive polymer block with conductive adhesive. The conductive polymer blocks with CNT/PDMS composite films attached are integrated together in PDMS housings to constitute a multidirectional force sensing tactile sensor.

The proposed sensor was fabricated in three different steps (Figure 2). The first step was to prepare thin CNT/PDMS composite films. A basic sensing element in the sensor is composed of two thin CNT/PDMS composite films having microdome structures on one side. The other side of the composite film is flat to be attached on the conductive polymer blocks of the central core or four sidewall structures. When two composite films contact each other, interlocking microdome structures are sandwiched making the electrical signal change. The CNT/PDMS mixture was prepared and casted on the silicon micromold in the reported way [19]. After evaporating solvent of the mixture in a vacuum desiccator, the composite film was fully cured at 90 °C for 3 h and detached from the micromold.

Second step was to fabricate conductive polymer blocks and attach thin CNT/PDMS composite films to them. Conductive polymer blocks were used as electrodes for sensing elements with its good physical flexibility and electrical conductivity. Metal molds having rectangular holes were manufactured by precision machining process. The size of holes and the thickness of the metal molds determine the size and thickness of central core’s middle and cap blocks and those of sidewall blocks. The holes were filled with conductive polymer (SCC conductive silicone, Sunkyoung S.T Co., Ltd., Hwaseong, Korea). The conductive polymer was cured on hot plate at 160 °C for 15 min and detached from the metal molds. Liquid phase conductive silicone adhesive (Loctite Ablestik ICP 4001, Henkel AG & Co. KGaA, Düsseldorf, Germany) was applied between the flat surface of the thin CNT/PDMS composite film and the conductive polymer block and cured on hot plate at 140 °C for 35 min.

Third step is to integrate the prepared central core and four sidewall structures with a PDMS housing. Polymer molds for the PDMS housing were first formed by using a 3D printer (Objet30 Pro, Stratasys, Ltd., Eden Prairie, MN, USA) and coated with 80 nm thick platinum layer by using a sputtering system to prevent swelling effect of the polymer molds during casting process. Prior to casting PDMS on the molds, the polymer molds were submerges in the polytetrafluoroethylene (PTFE) solution for 1 h and dried in room temperature for additional 1 h to prevent stiction between the mold and the cured PDMS. The bottom housing has flexible posts of the central core and empty rooms to place the four sidewall structures, while top housing has the room for the cap block of the central core. Six electrical lines were inserted into each conductive polymer block by using small needle. The conductive polymer block and its electrical line of the central core’s middle block acts as a common reference. The resistance of each sensing element is measured by two probe method between this common reference electrical line and five other electrical lines. Finally, both PDMS housings were secured together by curing PDMS which was inserted between the surfaces of two PDMS housings.

**Figure 3:**
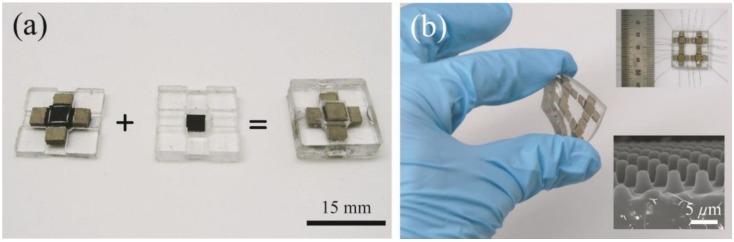
Photographs and scanning electron microscopic (SEM) image of the fabricated sensors (**a**) the bottom housing with the central core and four sidewall structures is assembled with the top housing to construct a sensor; (**b**) a 2 × 2 array sensor shows its flexibility. The embedded sensor image with a ruler shows the electrical lines inserted in the conductive polymer blocks. The embedded SEM image shows the surface of a CNT/PDMS composite film with microdome structures.

Figure 3 shows the fabricated tactile sensor. The transparent areas represent the PDMS housing while the light brown areas and the white areas are the conductive polymer blocks and conductive adhesive, respectively. Black areas are the thin CNT/PDMS composite films. The central core is positioned in the middle of the sensor and surrounded by the four sidewall structures. In Figure 3a, the black area shown in the center of top PDMS housing (middle) is the thin CNT/PDMS composite film on the bottom face of the cap block and the black area in the center of the bottom PDMS housing (left) is the thin CNT/PDMS composite film on the top face of the central core’s middle block. Overall there are six separate conductive polymer blocks; the middle block and the cap block of the central core and the four sidewall structures. The size of a single sensor device is 15 mm × 15 mm × 5 mm (W × D × H). Also 2 × 2 array type sensor is shown in Figure 3b. The fabrication process for an array type sensor is almost identical to those of single sensor device except the integration process with a PDMS housing.

## 4. Experimental Results

The sensor was characterized in three different ways. First, the response to normal pressure was measured. The resistance between middle block and cap block of the central core was measured while applying normal pressure on the top PDMS housing. Second, the response to shear force was measured by monitoring the resistance change between middle block and one of four sidewall structures while applying shear force on the top PDMS housing. Finally, the qualitative isolation or reduced crosstalk between normal and shear force sensing elements was observed.

The fabricated sensors were tested with a custom-made tactile sensor characterization instrument. The instrument is equipped with an exchangeable multi-axis load cell, a linear motor based X-Y stage, and a signal acquisition system measuring the resistance of sensing elements. An installed load cell can move up-and-down in Z-direction. The instrument can be operated in constant force mode such that constant normal and shear force can be applied on the sensor.

**Figure 4:**
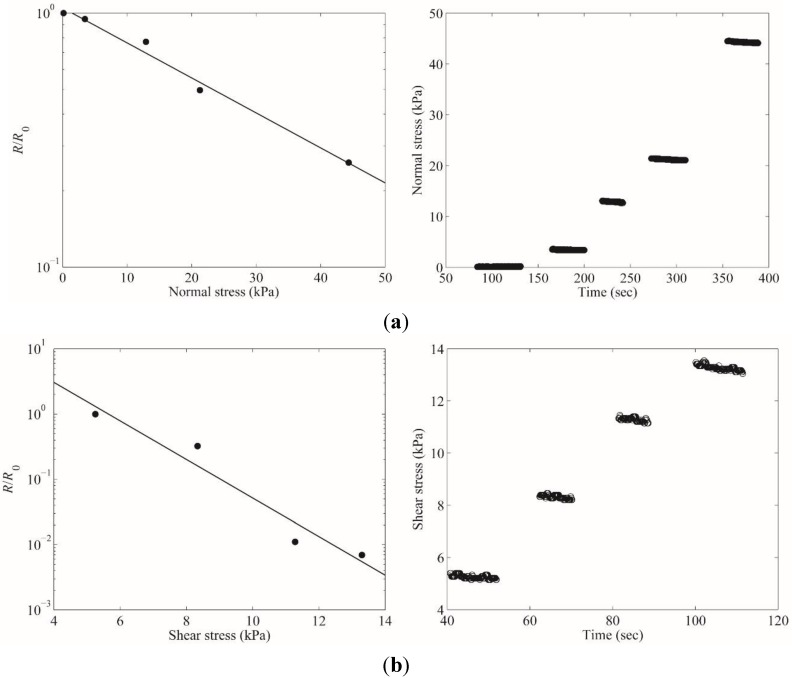
Response to normal and shear stress of the tactile sensor: (**a**) the resistance change in normal pressure sensing element under 128 Pa ~ 44 kPa normal forces; (**b**) the resistance change in shear stress sensing element under 5.28 kPa ~ 12.9 kPa shear forces.

The response to normal pressure was measured with the custom-made tactile sensor characterization instrument. Different amount of normal forces were applied on the top of the sensor and the resistance was measured between the electrical lines from the central core’s middle conductive polymer block and the cap conductive polymer block. The external force was applied on 4 mm × 4 mm with precise force control. Figure 4a shows that the resistance of the normal sensing element decreases as the applied force increases. The experimental results showed that the sensor can measure applied normal pressure in the range of 128 Pa ~ 44 kPa.

The response to a shear force was also measured with the tactile sensor characterization instrument. Similar to normal pressure measurement, the shear force was applied on the top of the sensor and the resistance between the common reference electrical line and the sidewall conductive polymer electrical line was recorded. Figure 4b demonstrates the resistance change of the shear force sensing element. The minimum shear force measurable was 5.28 kPa. The sensitivities measured were 0.165 k∙Pa^−1^ for shear direction and 0.0173 k∙Pa^−1^ for normal direction.

**Figure 5:**
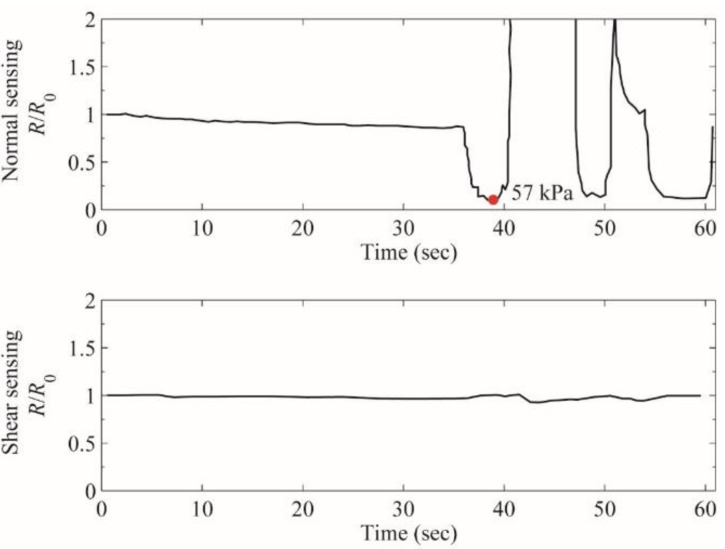
The resistance changes in normal pressure sensing element (top) and shear force sensing element (bottom): upon varying pure external normal force during 35 ~ 60 s, the resistance of normal pressure sensing element changes drastically following the amount of normal pressure applied while there was little disturbance in the resistance of shear force sensing element.

The unique characteristic of the fabricated sensor is its reduced crosstalk between the normal pressure sensing element and the shear force sensing element. Figure 5 shows the resistance of normal pressure sensing element (top graph) and shear force sensing element (bottom graph) upon pure external normal pressure. The graphs show clear discrimination of normal pressure and shear force into the resistance change of different sensing elements without interference. Pure normal pressure barely changed the resistance in the shear force sensing element while the resistance in normal pressure sensing element changes dramatically.

The demonstration of the capability of the 2 × 2 array type sensor in analyzing normal and shear force was carried out by using a LabVIEW-controlled 20 channels system. The demonstration system consists of voltage dividers, operational amplifiers, 12 bit 8-channel ADCs, a communication module and a microcontroller. When the resistance changes in each sensing element, its value is translated to voltage by the voltage divider, buffered and amplified by the operational amplifier. Then, the amplified analog signal is converted to a digital signal by the ADC and processed in a microcontroller to be displayed on a computer screen. As the resistance decreases, the color on the screen for each sensing element changes from green to red.

**Figure 6:**
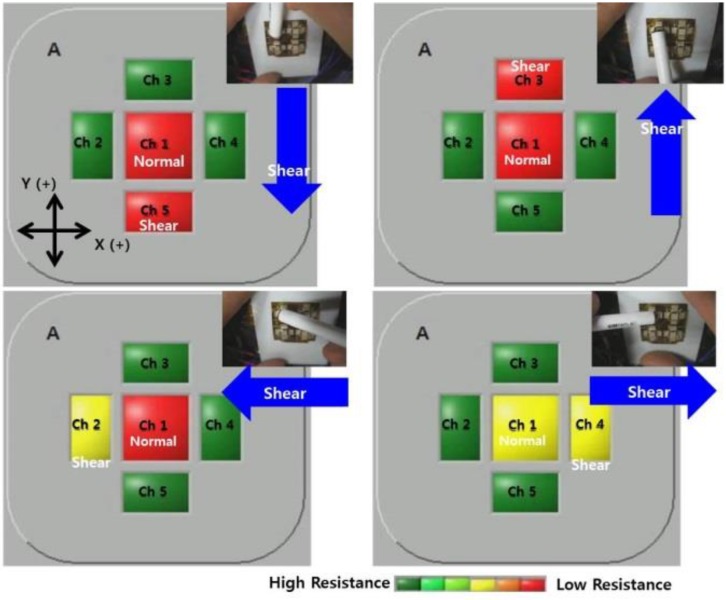
Screen capture of LabVIEW-controlled demonstration system to show the capability of the sensor in discriminating of applied shear force direction.

Figure 6 demonstrates the discrimination of the applied shear force direction. Ch1 represents the resistance level of a normal pressure sensing element while Ch2 ~ Ch5 represent the resistance level of shear force sensing elements in four directions respectively (X(−), Y(+), X(+), Y(−)). When force is not applied, the resistance level of each sensing element stays high and the color on the display is green. As the force applied on the sensor increases, the resistance level of affected sensing elements is lowered and the color on the display changes from green to yellow and to red. The shear forces were applied in four different directions on X-Y plane (Y(−), Y(+), X(−), X(+)). Normal pressure was also applied to minimize the slip during the test. When shear force was applied in Y(−) direction, only the color of Ch5 changes from green to red (top left display in Figure 6). Similarly the color of Ch3 changes upon the shear force in Y(+) direction. The color of Ch2 and Ch4 changed only upon the external shear force in X(−) and X(+) directions, respectively. This demonstration suggests the simple way to detect the multidirectional force without complex signal processing as in the central nerve system concept [31].

## 5. Conclusions

Human skin can sense and distinguish different types of tactile stimulations with various types of mechanoreceptors. The transduced tactile information is conveyed on the separated nerve fiber toward central nerve system without complex signal processing. Inspired by the organization and function of human skin’s tactile sensing capability, a tactile sensor was developed to sense normal and shear force separately in different sensing elements without interference and any post signal processing. The developed sensor demonstrated its capability to sense normal and shear pressures as low as 128 Pa and 5.28 kPa, respectively. Also, there was little interference between the normal pressure sensing element and the shear force sensing element upon applying pure normal pressure. The demonstration system successfully showed that the fabricated sensor can discriminate the force direction and magnitude. The developed sensor can be applied in the development of the prosthetic arms, especially those requiring the restoration of tactile sensation with its high sensitivity to recognize even gentle touch (under a few kPa pressure) and unique shear sensing capability to enable easy detection of slipping motions of holding objects. Further research need to be followed on the optimization to increase the dynamic range and to enhance its sensitivity and the minimum detectable shear pressure and the miniaturization to be embeddable on artificial skin type structures.
